# Something about the Journal

**Published:** 1892-09

**Authors:** 


### *EDITORIAL.*

The office of THE JOURNAL is in rooms 43 and 44 Old Capitol Building.
Address communications relating to the Editorial Department to DR. L. B. GRANDY, Atlanta, Ga., Box 431.
Business communications should be addressed to DR. M. B. HUTCHINS, Atlanta, Ga.
All remittances should be made payable to "ATLANTA MEDICAL AND SURGICAL JOURNAL."
Articles for publication should reach this office not later than the 15th instant.
The editors are not responsible for views expressed by contributors.

### SOMETHING ABOUT THE JOURNAL.

As it is customary for publishers occasionally to say something about themselves, it may not be amiss for THE JOURNAL to follow their example just at this time.

Six months have passed since the present management took entire control, but the policy of making a Journal which would be pleasing to its subscribers was inaugurated nearly two years ago. At that time all "insert" advertising leaves were removed from the reading pages, at considerable loss of money, it is true, but to the great improvement of the appearance of THE JOURNAL. Next followed the exclusion of all advertisement, or reading, notices from the literary pages, rendering it possible for a reader to go through the entire sixty-four pages of reading matter without being "caught" by an advertisement notice. Following close upon these changes came the reduction of the subscription price to an even $2.00 per annum, shortly followed by unequalled premium offers and privileges to all cash subscribers.

Coming down to the present management: The form and appearance of THE JOURNAL have been improved, and, the best of all, the character of its literary contents has been raised to a degree of excellence (with apologies to the Managing Editor) which it had probably never before attained. Much of the original matter inserted, the greater part of it, is directly or indirectly paid for, thus helping to raise the standard. But original articles from any source, however apparently obscure, are never rejected if possessing an element of interest which is likely to please even one subscriber. Regularly accepted *original* articles are permitted to contain references to preparations advertised in THE JOURNAL, for things which the writer has found useful should have every legitimate opportunity of being pressed upon the attention of other physicians.

One of the best features of THE JOURNAL is its monthly New York letter, furnished by Dr. William L. Russell. Dr. Russell is one of the best medical correspondents in the country. He has access to the meetings of all the prominent medical societies in New York City, and, being a faithful, conscientious worker, is enabled to make his letters especially valuable and interesting.

In addition to the regular number of purely original articles, the managers of THE JOURNAL will furnish, from time to time, *translations* of the best medical articles to be found in the French and German publications. One of these, from the French, appears in this issue. This is an advantage which few journals offer.

There are two ways of conducting a publication. One, apparently the most popular, is to give the advertisements the greater prominence and to let the subscriber take what he can get. The other way is to issue a first-class, clean publication in which the reading and the advertising matter are kept separate, so that the reader may know the one from the other at a glance, and know that he can find each department under its own proper heading. Subscribers who endure the first kind of publication do so either because they can't get any better, or because they have become so inured to the imposition that they take it as a matter of course, but reserve the privilege of harboring a grudge against the advertiser who obtrudes himself in the wrong place. Many people who are "caught" by an advertisement take offence at the advertiser for having it in reading matter and resolve to have revenge by not patronizing that particular advertiser, and thus counterbalance the gain which such a location might produce.

Our policy is to conduct THE JOURNAL in such a manner as to please the subscriber, thus keeping him on our list, and to make the publication so attractive to new readers as to get them to become permanent subscribers. If you don't please your subscribers you can't keep them, and if you don't keep them you can't make your advertising department pay. So which is better: advertisments and reading "mixed," colored advertising leaves, in other words the Hostetter almanac style, on the one hand; or a clean, tasteful journal with no admixture of matter or colors, after the style of the best literary magazines?

The number of *pages* of new subscribers, received this year, on our books demonstrates how well THE JOURNAL takes with the people.

The fact that not over three or four of the physicians who were asked to subscribe at the last meeting of the State Medical Association declined is further evidence of THE JOURNAL'S merits.

Advertisements are by no means neglected. They receive as much care and attention as the reading matter. Every legitimate effort is made to give the advertiser what he pays for, and the policy of THE JOURNAL towards advertisers is one of absolute fairness and honesty.

The stock of THE JOURNAL has increased in value about twenty-five per cent. in the past three years—so the policy which we have followed must "pay."

Comparison demonstrates that we are publishing the best two dollar journal in the South, giving our readers (64) sixty-four pages of clean reading matter each month, while others at this price have from (42) forty-two to (48) forty-eight pages, throughout which you find more or less advertising matter.

The Managing Editor of THE JOURNAL is Demonstrator of Anatomy in the Southern Medical College, while the Business Manager holds the same position in the Atlanta Medical College. This coincidence of positions will not only result in benefit to THE JOURNAL (as these positions bring the holders into most intimate contact with the students), but it will enable THE JOURNAL to render valuable assistance to the colleges, though remaining upon an absolutely neutral, non-partisan basis. THE JOURNAL will not be the organ of any college, or any person, but will maintain an attitude of neutrality and friendship to all, the proprietors acting as individuals where their interests lie and for THE JOURNAL where its interests lie.

